# CNS-LAND score: predicting early neurological deterioration after intravenous thrombolysis based on systemic responses and injury

**DOI:** 10.3389/fneur.2023.1266526

**Published:** 2023-09-21

**Authors:** Huijuan Jin, Rentang Bi, Yifan Zhou, Qinghui Xiao, Min Li, Shuai Sun, Jinghua Zhou, Jichuan Hu, Ming Huang, Yanan Li, Candong Hong, Shengcai Chen, Jiang Chang, Yan Wan, Bo Hu

**Affiliations:** ^1^Department of Neurology, Union Hospital, Tongji Medical College, Huazhong University of Science and Technology, Wuhan, China; ^2^Department of Neurology, The Second People’s Hospital of China Three Gorges University, Yichang, China; ^3^Department of Neurology, The First Clinical Medical College of China Three Gorges University, Yichang, China; ^4^Department of Neurology, People’s Hospital of Dongxihu District, Wuhan, China; ^5^Department of Neurology, Hubei Provincial Hospital of Integrated Chinese and Western Medicine, Wuhan, China; ^6^School of Public Health, Tongji Medical College, Huazhong University of Science and Technology, Wuhan, China

**Keywords:** early neurological deterioration, intravenous thrombolysis, acute ischemic stroke, scale, clinical interventions

## Abstract

**Importance:**

Early neurological deterioration (END) is a critical complication in acute ischemic stroke (AIS) patients receiving intravenous thrombolysis (IVT), with a need for reliable prediction tools to guide clinical interventions.

**Objective:**

This study aimed to develop and validate a rating scale, utilizing clinical variables and multisystem laboratory evaluation, to predict END after IVT.

**Design, setting, and participants:**

The Clinical Trial of Revascularization Treatment for Acute Ischemic Stroke (TRAIS) cohort enrolled consecutive AIS patients from 14 stroke centers in China (Jan 2018 to Jun 2022).

**Outcomes:**

END defined as NIHSS score increase >4 points or death within 24 h of stroke onset.

**Results:**

1,213 patients (751 in the derivation cohort, 462 in the validation cohort) were included. The CNS-LAND score, a 9-point scale comprising seven variables (CK-MB, NIHSS score, systolic blood pressure, LDH, ALT, neutrophil, and D-dimer), demonstrated excellent differentiation of END (derivation cohort C statistic: 0.862; 95% CI: 0.796–0.928) and successful external validation (validation cohort C statistic: 0.851; 95% CI: 0.814–0.882). Risk stratification showed END risks of 2.1% vs. 29.5% (derivation cohort) and 2.6% vs. 31.2% (validation cohort) for scores 0–3 and 4–9, respectively.

**Conclusion:**

CNS-LAND score is a reliable predictor of END risk in AIS patients receiving IVT.

## Introduction

Intravenous thrombolysis (IVT) with r-tPA is an effective approach for vascular recanalization in patients with acute ischemic stroke (AIS), but around 6.5 to 7% of patients experience early neurological deterioration (END) after IVT. Numerous studies have demonstrated that END is strongly associated with mortality and major disability (1–4). To prevent long-term disability or death, early interventions such as dehydration, antiplatelet, anti-inflammatory, bridging therapy, and decompressive craniectomy may be necessary. Rapid prediction of END is important not only for making timely clinical decisions and improving patient outcomes, but also for conserving healthcare resources and maintaining a positive physician-patient relationship. However, identifying patients with a high risk of END is challenging. END can be unpredictable even for experienced neurologists, as some patients may initially have only mild neurological deficits (5, 6).

Although END has become an increasingly attractive therapeutic target in post-stroke treatment, its exact underlying pathophysiological mechanism remains unclear (7–9). Except for symptomatic intracerebral hemorrhage, malignant vasogenic cerebral edema, thrombus expansion, and early secondary epilepsy, there seems to be no unmasked culprit for most cases of END (10). Strikingly, the autonomic dysfunction, the hypothalamic–pituitary–adrenal (HPA) axis, immunosuppression, and damage-associated molecular patterns (DAMPs) from the brain, account for multi-system complications immediately after ischemic stroke (11, 12). Meanwhile, systemic responses and injuries to remote organs substantially impact the progression and outcome of cerebral ischemic injury (13, 14). Epidemiological studies have accumulated strong evidence indicating that multiple organ dysfunction promote neurological deterioration, and that serum or organ-specific biomarkers were significantly associated with END risk (15, 16), despite a lack of validation in IVT patients.

Combining clinical variables with multisystem hematological indicators holds promise to improve risk stratification for END. Actually, recent studies suggest that patients with clinical features such as severe neurological deficits, high blood pressure, high blood glucose, high neutrophil/lymphocyte ratio, prior aspirin administration, and neuroimaging features such as middle cerebral artery density, early CT ischemic lesions are considered more prone to END after IVT (17–19). However, the limited sample and inconsistencies between studies imply the unreliability of these conclusions. To date, no END risk prediction model has been developed that combines clinical variables and multisystem laboratory evaluation.

The aim of this study is to develop and externally validate a scoring model for identifying patients with a high risk of END after IVT.

## Methods

This study was conducted in accordance with the principles outlined in the Declaration of Helsinki on medical ethics and was approved by the local institutional review board (ChiCTR2000033456).

### Study design and data sources

The Multicenter Clinical Trial of Revascularization Treatment for Acute Ischemic Stroke (TRAIS) study retrospectively collected data from all consecutive patients with acute stroke admitted to 14 Chinese stroke centers, including the Wuhan Union hospital, the Wuhan Union Hospital West Campus, the Wuhan Union Hospital Jinyinhu Campus, the People’s Hospital of Dongxihu District, the Hubei Provincial Hospital of Integrated Chinese and Western Medicine, the Central People’s Hospital of Yichang, the First People’s Hospital of Yichang, the Second People’s Hospital of Yichang, the Central Hospital of Hefeng County, the People’s Hospital of Honghu, the People’s Hospital of Jingshan, the First People’s Hospital of Jiangxia District, Wuhan Red Cross Hospital, Puren Hospital of Wuhan.

Patients were included in the study if they met the following criteria: (1) retrospective clinically and radiologic confirmed AIS; (2) received rt-PA injections according to the indications for thrombolytic therapy; and (3) were aged 18 years or older. Exclusion criteria included: (1) mental disorders or severe cognitive dysfunction; and (2) incomplete clinical data. All centers follow the same patient screening process.

The analysis included patients admitted between January 1, 2018 and June 1, 2021. All patients were informed of their participation in the study.

### Clinical and radiological data

We collected the following data: (1) clinical assessments, including the National Institute of Health Stroke Scale (NIHSS) score evaluated on admission and 24 h after thrombolysis, baseline blood pressure, and time from onset to treatment (OTT); (2) demographic information, such as gender and age; (3) vascular risk factors, including alcohol consumption, smoking history, hypertension, diabetes mellitus, coronary heart disease, and previous stroke; (4) laboratory tests, including admission blood glucose level, C-reaction protein (CRP), white blood cell count (WBC), red blood cell count (RBC), neutrophil count, lymphocyte count, platelet count (PLT), D-dimer, international normalized ratio (INR), prothrombin time (PT), activated partial thromboplastin time (APTT), fibrinogen (FIB), total bilirubin (TBil), direct bilirubin (DBil), glutamic pyruvic transaminase (ALT), glutamic oxiracetam transaminase (AST), blood urea nitrogen (BUN), creatinine (Cr), uric acid (UA), lactic dehydrogenase (LDH), creatine kinase (CK), creatine kinase myocardial band (CK-MB), cardiac troponin I (cTNI).

### Definition of END

According to the European Cooperative Acute Stroke Study, END was defined as an increase in NIHSS score of 4 or more points or death within 24 h (20).

### Statistical analysis

Categorical variables were reported as counts and percentages, while continuous variables were presented as means and standard deviations. We used ordinal logistic regression to calculate the odds ratio (OR) and 95% confidence interval (CI) for patients with and without END in the derivation cohort. Briefly, to assess the univariate relationship between baseline variables and END, we used t-tests or Mann–Whitney U tests for continuous variables, and χ^2^ or Fisher exact test for categorical variables, as appropriate. With the dependent variable END, we conducted a stepwise multivariable binary logistic regression analysis. We used the C statistic to assess the discriminative ability of the score in predicting END. We used SAS version 9.4 (SAS Institute) and SPSS version 16.0 (SPSS Inc.) for statistical analysis. We considered statistics to be significant at a two-tailed *p*-value of less than 0.05.

## Results

### Patient characteristics

**Figure 1:**
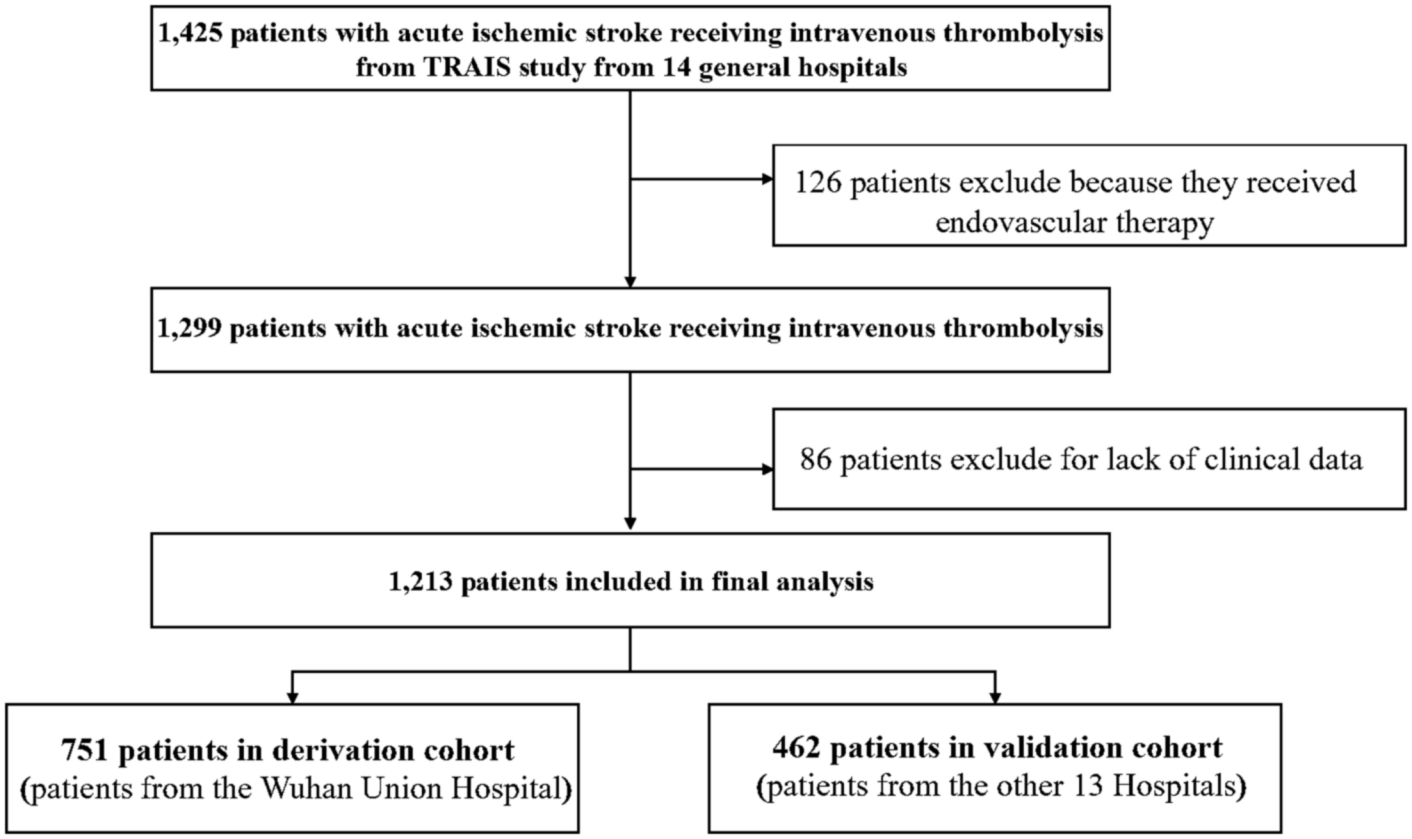
Flowchart of patient inclusion in the study. A total of 1,425 patients with acute ischemic stroke (AIS) received intravenous thrombolysis (IVT) in the TRAIS study. Of these, 126 patients were excluded because they received endovascular therapy. After excluding an additional 86 patients due to lack of clinical data, a total of 1,213 patients were included in the final analysis. The patients were divided into two cohorts: a derivation cohort consisting of 751 patients from Wuhan Union Hospital, and a validation cohort consisting of 462 patients from 13 other hospitals.

Between January 2018 and June 2022, 1,425 patients with AIS were treated with IVT in the TRAIS study. Of these, 212 patients were excluded (126 received endovascular therapy and 86 patients lacked clinical data), and the remaining 1,213 patients were included in the final analysis (Figure 1). We set the patients in Wuhan Union Hospital as the training cohort, and the patients in other 13 medical centers as the validation cohort. The derivation cohort consisted of 751 patients, while the validation cohort consisted of 462 patients. In the derivation cohort, 487 (63.4%) patients were male, the mean age (±SD) was 64.6 (±12.5) years, and the median NIHSS (interquartile range [IQR]) score was 3 (1–7). In the validation cohort, 315 (67.9%) patients were male, the mean age (±SD) was 66.9 (±11.8) years, and the median NIHSS (IQR) score was 5 (3–10). Patients in the derivation cohort had a higher prevalence of cigarette consumption, lower frequency of previous stroke, ischemic heart disease, hyperlipidemia, and lower admission NIHSS score. Table 1 provides detailed baseline characteristics of patients in the derivation and validation cohort.

**Table 1:** Baseline characteristics and outcomes of the Study.

Characteristics	Total cohort (*N* = 1,213)	Training cohort (*N* = 751)	Validation cohort (*N* = 462)
Demographic characteristics			
Age, years	65.5 (±12.3)	64.6 (±12.5)	66.9 (±11.8)
Male	809 (66.0)	487 (63.4)	315 (67.9)
Medical history			
smoking	392 (31.8)	277 (36.1)	115 (24.8)
alcohol	235 (19.1)	150 (19.5)	85 (18.9)
Ischemic stroke	171 (13.9)	102 (13.3)	69 (14.9)
Intracerebral hemorrhage	32 (2.6)	15 (2.0)	17 (3.7)
Ischemic heart disease	156 (12.7)	92 (12.0)	81 (17.5)
Hypertension	730 (59.3)	457 (59.5)	273 (58.8)
Hyperlipidemia	289 (23.5)	192 (25.0)	107 (23.1)
Diabetes	265 (21.5)	158 (20.6)	97 (20.9)
Admission characteristics			
OTT, min	180 (126–240)	208 (148–260)	150 (105–194)
SBP, mm Hg	148 (133–161)	146 (133–160)	150 (134–166)
Baseline NIHSS	4 (2–9)	3 (1–7)	5 (3–10)
Admission laboratory values			
Glucose	6.6 (5.6–8.3)	6.6 (5.6–8.2)	6.7 (5.6–8.6)
CRP, ×109/L	1.72 (0.79–4.37)	1.71 (0.81–4.11)	1.76 (0.75–5.06)
WBC, ×109/L	6.86 (5.56–8.74)	6.70 (5.48–8.58)	7.16 (5.70–9.08)
RBC, ×109/L	4.48 (4.13–4.84)	4.51 (4.11–4.94)	4.43 (4.09–4.78)
PLT, ×10 9/L	193 (159–233)	191 (159–236)	197 (158–230)
NEU, %	72.8 (63.8–80.8)	72.5 (63.7–80.6)	73.6 (63.6–81.0)
LYM, %	19.4 (12.9–27.5)	19.9 (13.2–27.7)	17.3 (11.9–26.2)
Ddimer, mg/L	0.41 (0.27–0.87)	0.39 (0.27–0.74)	0.55 (0.26–1.65)
INR	12.8 (11.9–13.5)	13.1 (12.7–13.7)	11.4 (10.6–12.7)
PT, s	1.01 (0.96–1.08)	1.01 (0.96–1.07)	1.02 (0.96–1.1)
APTT, s	33.5 (30.5–36.8)	34.9 (32.4–37.6)	29.9 (26.7–33.1)
FIB, g/L	3.22 (2.74–3.77)	3.25 (2.77–3.73)	3.22 (2.4–4.28)
Tbil, μmol/L	10.3 (7.7–14.3)	10.2 (7.6–14.1)	10.9 (7.8–14.6)
Dbil, μmol/L	3.6 (2.6–5.1)	4 (2.9–5.5)	2.5 (1.6–3.9)
ALT, U/L	17 (12–24)	17 (12–25)	17 (11–24)
AST, U/L	20 (16–25)	20 (16–25)	21 (17–27)
BUN, mmol/L	5.7 (4.7–7.0)	5.6 (4.5–6.8)	6.0 (4.9–7.6)
Cr, μmol/L	77.0 (66.5–95.0)	76.3 (66.8–93.7)	78 (66–97)
UA, μmol/L	362 (296–448)	364 (298–440)	361 (296–454)
CK, U/L	87 (61–126)	85 (61–124)	94 (61–144)
LDH, U/L	187 (163–220)	190 (164–226)	181 (157–220)
CK-MB, ng/mL	1.2 (0.8–3.1)	1.1 (0.8–1.6)	2.4 (1.7–3.0)
cTNI, ng/L	3.4 (1.6–8.2)	4.1 (2.2–9.3)	2.8 (0.6–5.5)
Outcome			
END	101 (8.2)	49 (6.5)	44 (9.5)

Continuous and ordinal variables were reported as median (IQR), and categorical variables were presented as *n* (%). OTT, onset to treatment time; SBP, systolic blood pressure; FBG, fasting blood-glucose; CRP, C-reactive protein; WBC, white blood cell count; RBC, red blood cell count; PT, prothrombin time; INR, international normalized ratio; APTT, activated partial thromboplastin time; FIB, fibrinogen; TBIL, total bilirubin; DBIL, direct bilirubin; ALT, alanine transaminase; AST, aspartate aminotransferase; BUN, blood urea nitrogen; Cr, creatinine; UA, uric acid; LDH, lactate dehydrogenase; CK, creatine kinase; CK-MB, creatine kinase MB isoenzyme; cTNI, cardiac troponin I; END, early neurological deterioration. *p* value < 0.05 was represented in bold type.

### Univariate analysis of associations with END after IVT

Table 2 displays the results of the univariate analysis of variables related to END in the derivation cohort. Out of the derivation cohort, 49 patients (7.3%) experienced END. Among the patients who experienced END, 29 (51.8%) were male. The median NIHSS (IQR) score for these patients was 13 (6–21). To facilitate clinical application, we classified continuous and sequential variables and selected cut-off values based on common clinical values and actual results. In the univariate analysis, cigarette smoking, higher admission systolic blood pressure, baseline NIHSS score, white blood cell count, neutrophil ratio, D-dimer, and low lymphocyte ratio were found to be significantly correlated with END. Diabetes, lower, and higher AST were also associated weak to moderately associated with END.

**Table 2:** Univariate analysis comparing patients with and without END in derivation cohort.

	END (*N* = 49)	Non-END (*N* = 702)	*P* value
Demographic characteristics			
Age > 60, years	38 (67.9)	455 (63.9)	0.304
Male	29 (51.8)	458 (64.3)	0.126
Medical history			
smoking	684 (66.0)	266 (37.4)	**0.008**
alcohol	9 (16.1)	141 (19.8)	0.498
Ischemic stroke	102 (9.8)	54 (7.6)	0.077
Intracerebral hemorrhage	42 (4.1)	9 (1.3)	0.398
Ischemic heart disease	672 (64.8)	56 (7.9)	0.451
Hypertension	20 (1.9)	428 (60.1)	0.222
Hyperlipidemia	101 (9.7)	107 (15)	0.213
Diabetes	5 (8.9)	153 (21.5)	**0.025**
Admission characteristics			
OTT, min	5 (8.9)	112 (15.7)	0.214
SBP>160, mm Hg	20 (35.7)	150 (21.1)	**0.011**
Baseline NIHSS			
0–4	10 (17.9)	450 (63.2)	<0.001
5–15	17 (30.4)	220 (30.9)	
16–42	22 (39.3)	32 (4.5)	
Admission laboratory values			
Glucose ≥7.0	16 (28.6)	176 (24.7)	0.114
CRP ≥8, ×109/L	13 (23.2)	116 (16.3)	0.143
WBC ≥ 10, ×109/L	9 (16.1)	98 (13.8)	**0.011**
RBC <3.8, ×109/L	7 (12.5)	51 (7.2)	0.103
NEU ≥80, %	45 (80.4)	404 (56.7)	**0.001**
LYM <20, %	29 (51.8)	266 (37.4)	**0.015**
Ddimer ≥0.5, mg/L	27 (48.2)	202 (28.4)	**0.001**
INR ≥1.31	4 (7.1)	11 (1.5)	**0.003**
PT ≥16, s	4 (7.1)	13 (1.8)	**0.007**
APTT ≥43.5, s	3 (5.4)	25 (3.5)	0.985
FIB ≥4, g/L	9 (16.1)	88 (12.4)	0.482
Tbil ≥22, μmol/L	8 (14.3)	49 (6.9)	**0.037**
Dbil ≥5, μmol/L	11 (19.6)	80 (11.2)	0.054
ALT ≥35, U/L	11 (19.6)	58 (8.1)	**0.003**
AST ≥40, U/L	6 (10.7)	31 (4.4)	**0.026**
BUN ≥8.2, mmol/L	7 (12.5)	62 (8.7)	0.302
Cr ≥106, μmol/L	8 (14.3)	96 (13.5)	0.805
UA ≥357, μmol/L	28 (50)	308 (43.3)	0.221
CK ≥174, U/L	9 (16.1)	103 (14.5)	0.765
LDH ≥220, U/L	30 (53.6)	152 (21.3)	<0.001
CK-MB ≥5, ng/mL	10 (17.9)	33 (4.6)	<0.001
cTNI ≥26.2, ng/L	202 (19.5)	183 (17.0)	0.147

Continuous and ordinal variables were reported as median (IQR), and categorical variables were presented as *n* (%). *p* value < 0.05 was represented in bold type.

### Independent predictors of END after IVT

Multivariate logistic regression analysis was performed to identify the independent predictors of END after IVT. Variables that were found to be significant in the univariate analyses were entered simultaneously into the multivariate model. After adjusting for confounding factors, CK-MB ≥ 5 ng/mL, NIHSS score, SBP ≥ 160 mmHg, LDH ≥ 220 U/L, ALT≥40 U/L, Neutrophil ratio ≥ 80%, D-dimer≥0.5 mg/L were all independent predictors of END (as shown in Table 3).

**Table 3:** Multivariate analysis for factors associated with END in training cohort.

Predictor variable	aOR (95% CI)	*P* value
CK-MB ≥5, ng/mL	3.063 (1.152, 8.149)	0.025
NIHSS score		
0–4	ref	
5–15	2.419 (1.046, 5.598)	0.039
16–42	14.339 (5.535,37.145)	<0.001
SBP ≥160, mmHg	2.486 (1.222, 5.054)	0.012
LDH ≥220, U/L	2.562 (1.218, 5.388)	0.013
ALT ≥40, U/L	2.664 (1.068, 6.648)	0.036
Neutrophil ratio ≥ 80, %	2.879 (1.224, 6.771)	0.015
D-dimer ≥0.5, mg/L	2.684 (1.248, 5.773)	0.011

aOR (95% CI) indicates adjusted Odds Ratio (95% Confidence Interval).

### END prediction score: CNS-LAND

The CNS-LAND scores (CK-MB, NIHSS score, Systolic blood pressure, LDH, ALT, Neutrophil ratio, D-dimer) were developed based on the multivariate logistic analysis in the derivation cohort, incorporating seven independent predictors of END after IVT: CK-MB, NIHSS score, systolic blood pressure, LDH, ALT, neutrophil ratio, and D-dimer. The weight of each predictor was determined by the strength of its association with the beta coefficient, and points were assigned accordingly. The total score ranged from 0 to 9, with higher scores indicating a greater risk of END (refer to Table 4 for details).

**Table 4:** Determinants of the CNS-LAND score.

Predictors	Score
***C*K-MB**	
<5, ng/mL	0
≥5, ng/mL	1
***N*IHSS score**	
0–4	0
5–15	1
16–42	3
***S*BP**	
<160, mmHg	0
≥160, mmHg	1
***L*DH**	
<220, U/L	0
≥220, U/L	1
***A*LT**	
<40, U/L	0
≥40, U/L	1
***N*EU**	
<80, %	0
≥80, %	1
***D*-dimer**	
<5 ng/mL	0
≥5 ng/mL	1
Total Score	9

In both the derivation and validation cohorts, there was a significant increase in the proportion of patients with END as the CNS-LAND score increased, as shown in Table 5. To facilitate clinical application, we classified patients with low risk of functional dependence as having a score of 0–3 and those with high risk as having a score of 4–9. The cut-off value was determined from the receiver operating characteristic (ROC) curve analysis to achieve optimal performance. In the derivation cohort, 122 patients (16.2%) and in the validation cohort, 112 patients (81.2%) had a score ≥ 4, with a predicted sensitivity of 73.5 and 72.7%, respectively, and a predicted specificity of 87.7 and 80.8%, respectively. Further details of the classification test are provided in Table 5.

**Table 5:** Proportion of patients with END stratified by CNS-LAND score.

	Training cohort (*N* = 751)	Validation cohort (*N* = 462)
C-statistics (95% CI)	0.862 (0.796–0.928)	0.900 (0.849–0.950)
Score
0	2/91 (2.2)	0/23 (0)
1	2/235 (0.9)	0/90 (0)
2	3/198 (1.5)	4/126 (3.2)
3	6/105 (5.7)	8/111 (7.2)
4	8/62 (12.9)	9/60 (15)
5	11/33 (33.3)	6/27 (22.2)
6	10/17 (58.8)	12/18 (66.6)
7	3/6 (50)	3/5 (60)
8	4/4 (100)	2/2 (100)
Dichotomized score		
0–3	13/629 (2.1)	12/350 (3.4)
4–9	36/122 (29.5)	32/112 (28.8)
Dichotomized test characteristics (95% CI)		
Sensitivity	0.735 (0.587–0.846)	0.727 (0.570–0.845)
Specificity	0.877 (0.850–0.900)	0.809 (0.766–0.844)
PPV	0.295 (0.218–0.386)	0.286 (0.206–0.380)
NPV	0.979 (0.964–0.988)	0.966 (0.939–0.981)
PLR	5.997 (4.625–7.776)	3.800 (2.908–4.965)
NLR	0.302 (0.190–0.482)	0.337 (0.207–0.547)

The proportion of patients was represented as *n*/*N* (%). CI, confidence interval; PPV, positive predictive value; NPV, negative predictive value; PLR, positive likelihood ratio; NLR, negative likelihood ratio.

The CNS-LAND score demonstrated good discrimination and calibration in both the derivation cohort (c statistic, 0.862; *p* value of Hosmer-Lemeshow test, 0.123) and the validation cohort (c statistic, 0.851; p value of Hosmer-Lemeshow test, 0.685).

### Comparing the CNS-LAND score with 3 existing AIS prognostic scores

**Figure 2:**
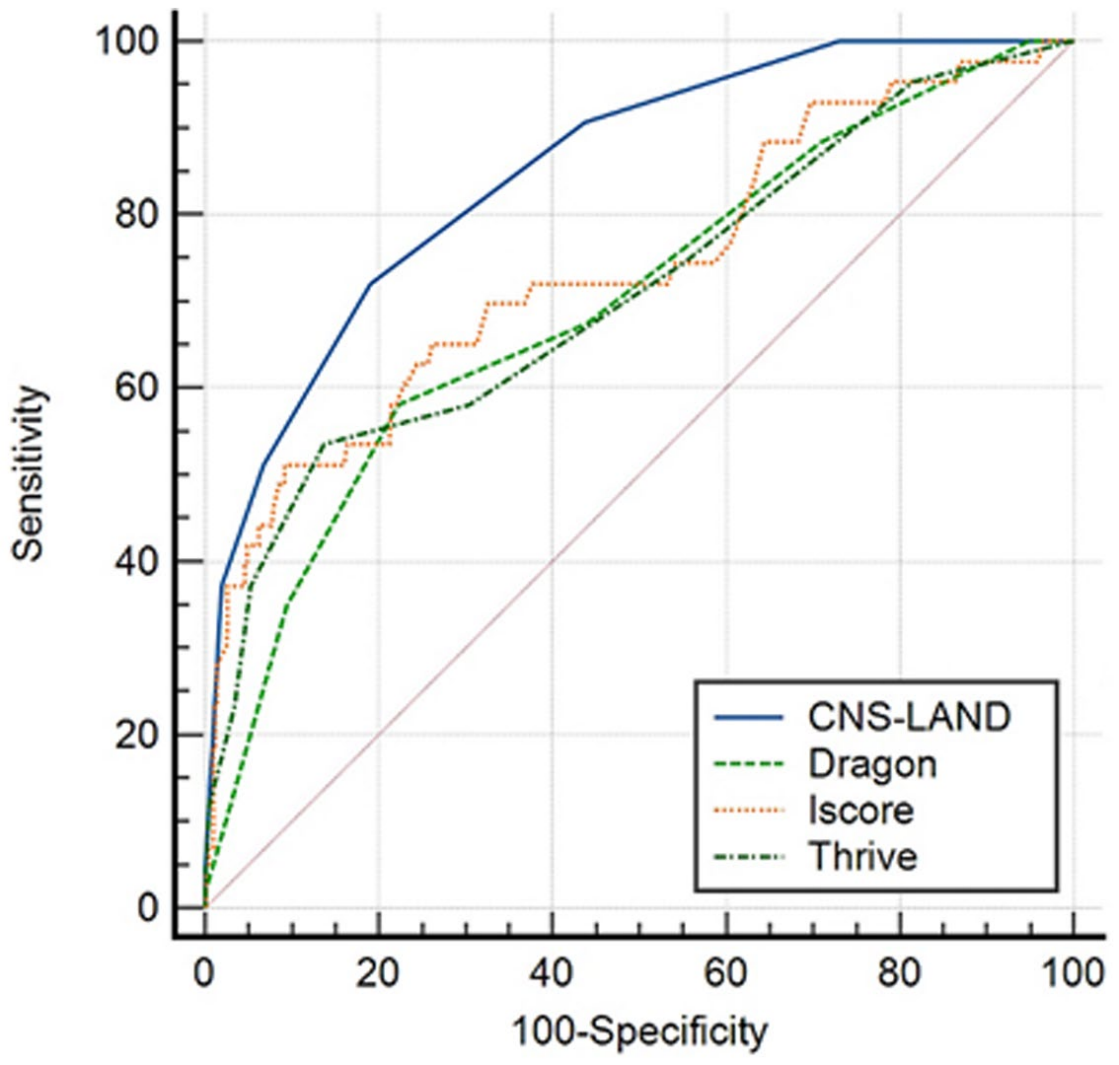
Receiver operating characteristic (ROC) curve comparing the discrimination ability of CNS-LAND and 3 existing AIS prognostic scores for predicting END. The analysis was based on the validation cohort. The ROC curve demonstrates the trade-off between sensitivity and specificity for each prediction score, with a larger AUC indicating better discrimination ability.

We compared the discriminative performance of the CND-LAND with three existing AIS prognostic scores including DRAGON, ISCORE, and THRIVE, in the validation cohort (*n* = 456). Six patients were excluded from the analysis due to missing information on elements of other scores. For END, the area under the curves (AUCs) ranged from 0.700 to 0.851 (DRAGON score, 0.700; THRIVE, 0.710; ISCORE score, 0.739; CNS-LAND score, 0.851). The CNS-LAND score had the highest AUC. The pairwise difference in AUCs between the CNS-LAND and other scores showed statistical significance (all *p* < 0.001) (see Supplementary Table S1). Figure 2 displays the ROC curves of the aforementioned scores for END.

## Discussion

This study demonstrated several important findings: (1) CK-MB ≥ 5 ng/mL, NIHSS score, SBP ≥ 160 mmHg, LDH ≥ 220 U/L, ALT ≥40 U/L, Neutrophil ratio ≥ 80%, and D-dimer ≥0.5 mg/L were identified as independent predictors of END, highlighting the relationship between systemic response and early stroke progression after IVT; (2) The CNS-LAND score showed good discrimination and calibration and was successfully validated in an independent cohort.

### Independent predictors of END

Research on peripheral biomarkers for END risks remains relatively limited, although AIS is widely recognized as a systemic disease that initiates excitotoxicity, neuroinflammation, and oxidative stress in the brain parenchyma, leading to peripheral immune disorders, autonomic nervous disorders, neuroendocrine overstimulation, and even damage to distant organs (11–14, 16). In this study, we assessed and validated the association between biomarkers reflecting excessive neuroinflammation, coagulation abnormalities, hemorheological disorders, and remote organ injury, and END risks.

Excessive neuroinflammation has been identified as a significant cause of stroke progression after IVT. Following AIS, neuronal death and microglial activation release large amounts of DAMPs and chemokines, which activate and recruit peripheral immune cells. Neutrophils, with their strong pro-inflammatory phenotype, are the first immune cells to infiltrate the brain (21, 22). They release not only pro-inflammatory cytokines and reactive oxygen species that cause secondary neuronal damage, but also matrixmetalloproteinase 9, which damages the blood–brain barrier, leading to acute vasogenic cerebral edema (23, 24). Moreover, neutrophil adhering to the microvascular walls can block the flow of red blood cells, resulting in the no-reflow phenomenon in the brain (25). Additionally, the excessive activation of neutrophils causes immunodepletion, making the host susceptible to secondary infections (26). These factors collectively contribute to a sharp increase in END risk.

D-dimer is general activated in response to thrombotic events, including AIS, acute myocardial infarction (AMI), pulmonary embolism, and venous thromboembolism (27). Recent studies have confirmed its association with stroke progression and early mortality (28). In studies on post-stroke complications during hospitalization, D-dimer levels are used for the early prediction of stroke recurrence, acute decompensated heart failure and respiratory tract infections (29–31). It is reasonable to speculate that elevated D-dimers not only contributed to cerebral thrombus expansion, but also increase the risk of thrombosis in the heart, liver, and even throughout the body, resulting in END.

Elevated systolic blood pressure may indicate a disorder of vascular autoregulation (3, 32–34). High systolic blood pressure can cause vasodilation of the microvessels and hyperperfusion in the infarct area, leading to cerebral edema and blood–brain barrier damage (35, 36). Currently, the management of blood pressure after acute ischemic attack is controversial. We found that admission systolic blood pressure ≥ 160 mmHg was significantly associated with END after IVT, supporting the notion that active intervention of blood pressure at admission improves patient outcomes.

Moreover, we identified novel END independent predictors: CK-MB, LDH, and ALT, which represent specific damage to distant organs after stroke. Previous studies have shown that AIS often results in cardiac abnormalities such as arrhythmias, autonomic nervous disorders, AMI, and paroxysmal arterial hypertension, which cause secondary damage to the brain. Heart failure or arrhythmias such as atrial fibrillation and flutter may lead to blood stasis in the left atrium, resulting in cardiogenic stroke (13, 14). It has been found that 4.9% of AIS patients experienced AMI, with worse outcomes than patients without AMI, and elevated cardiac parameters were associated with functional recovery at 3 months and 1 year in patients with AIS (37). Here, CK-MB and LDH, as traditional cardiac markers, were found to be associated with END risk. In addition, LDH may originate from ischemic cerebral hemispheres in addition to the myocardium. Intracellular LDH is upregulated for energy utilization and adaption to the ischemia in all injured brain cell, which was released into the extracellular space and then into the peripheral circulation through the damaged BBB. Our previous research has validated that serum LDH levels are associated with cerebral infarct size and cerebral edema and predicted neurological changes and 90-day outcomes (38).

Following stroke, chemokines CXCL1and CCL2 levels are elevated in liver, which lead to an increase in neutrophils and monocytes and an excessive inflammatory response, resulting in the release of ALT from necrotic hepatocytes. At the same time, brain-derived glutamate toxicity also promotes the synthesis of ALT, which metabolizes blood glutamate to provide protection. But more importantly, ALT has been found to be associated with symptomatic intracranial hemorrhage and mortality after AIS in recent studies (39, 40). Liver dysfunction (including fibrosis and cirrhosis), significantly impaired platelet aggregation, and diminished antifibrinolytic activity are hypothesized to cause the syndrome. In other words, ALT elevation reflects reduced liver function and damaged coagulation system, resulting in hemorrhagic transformation and END after IVT.

In conclusion, our study has identified several novel biomarkers associated with END risks, including CK-MB, LDH, ALT, D-dimer, neutrophil ratio, NIHSS score, and SBP. These biomarkers reflect different pathological processes, including cardiac damage, remote organ injury, excessive neuroinflammation, coagulation abnormalities, and hemorheological disorders, which could contribute to the development of END. Further studies are needed to validate these biomarkers’ clinical significance in predicting END risks and guide personalized treatment strategies.

### CNS-LAND score

CNS-LAND score is the first scale for predicting the END after IVT based on systemic injury and responses. With increasing CND-LAND scores, the risk at the END was gradually increased in the derivation and validation cohort. The score is generally divided into two categories: low-risk patients (0–3 points) who can benefit from reduced medical costs, and high-risk patients (4–9 points) who require multiple interventions based on close follow-up monitoring.

Currently, there are several scales based on large sample, multi-center cohorts for evaluating the hemorrhagic transformation risks and 3-month prognosis of patients with AIS, such as the DRAGON score, ISCORE score, and THRIVE score. However, these scales have little external verification of their discriminant capacities for END risk. In comparison, CNS-LAND is superior to these scales in predicting END. First, CNS-LAND includes END-specific hematologic indicators and clinical features. Second, it is based on a large multicenter TRAIS study sample, with patients continuously recruited from large medical centers. Additionally, CNS-LAND is based on hematological examination and clinical characteristics that are widely used in routine practice, has a short turnaround time, and does not require special laboratory equipment, making it convenient for primary hospitals. Lastly, we verified the differential power and diagnostic accuracy of the score in an independent cohort of 426 patients recruited from 13 centers in Hubei Province, China. Therefore, CNS-LAND scoring is currently the most feasible and applicable in clinical settings in China.

### Limitations

The study has several limitations: (1) the inclusion of patients from only large hospitals in China introduces inevitable selection bias, as patients from primary care institutions were not included; (2) imaging information, such as the thrombus location, core infarction and ischemic penumbra was not analyzed, because these data were not routinely collected for AIS patients; (3) as an observational and retrospective study, confounding factors could not be completely eliminated, although baseline data were refined to the extent possible.

## Conclusion

The CNS-LAND score can accurately screen out patients who will still suffer from END after IVT, so as to assess whether they need further clinical intervention and monitoring.

## Data availability statement

The datasets presented in this article are not readily available because the raw data contains personal information. Requests to access the datasets should be directed to HJ, jinhuijuan1983@163.com; BH, hubo@mail.hust.edu.cn.

## Ethics statement

The study protocol was approved by the ethics committee of the Union Hospital, Tongji Medical College, Huazhong University of Science and Technology, Wuhan, China (ChiCTR2000033456). The studies were conducted in accordance with the local legislation and institutional requirements. The participants provided their written informed consent to participate in this study.

## Author contributions

HJ: data curation, funding acquisition, supervision, writing – original draft, writing – review and editing. RB: data curation, methodology, writing – original draft, writing – review and editing. YZ: methodology, writing – original draft, writing – review and editing. QX: investigation, writing – review and editing. ML: writing – original draft. SS: writing – original draft. JZ: data curation, writing – original draft. JH: writing – original draft. MH: writing – original draft. YL: writing – original draft. CH: writing – original draft. SC: writing – original draft. JC: data curation, writing – original draft, writing – review and editing. YW: writing – original draft, writing – review and editing. BH: data curation, funding acquisition, supervision, writing – original draft, writing – review and editing.

## Funding

The author(s) declare financial support was received for the research, authorship, and/or publication of this article. This work was supported by the National Natural Science Foundation of China (grants: 82090044 and 81820108010 to BH; 82171306 to HJ), the National Key Research and Development Program of China (2018YFC1312200 to BH).

## Conflict of interest

The authors declare that the research was conducted in the absence of any commercial or financial relationships that could be construed as a potential conflict of interest.

## Publisher’s note

All claims expressed in this article are solely those of the authors and do not necessarily represent those of their affiliated organizations, or those of the publisher, the editors and the reviewers. Any product that may be evaluated in this article, or claim that may be made by its manufacturer, is not guaranteed or endorsed by the publisher.
