# Supplementary material for: CNS-LAND score: predicting early neurological deterioration after intravenous thrombolysis based on systemic responses and injury
Source: Front Neurol. 2023 Sep 21;14:1266526. doi: 10.3389/fneur.2023.1266526 (PMC10552779; doi:10.3389/fneur.2023.1266526)
Supplement: Supplementary file 1 [file Table_1.docx]

Table S1 Pairwise comparison of ROC curves between CNS-LAND and 3 existing AIS prognostic scores

|  | Difference between areas | 95%CI | Significance level |
| --- | --- | --- | --- |
| CNS-LAND vs DRAGONE | 0.151 | 0.0808-0.221 | p＜0.001 |
| CNS-LAND vs ISCORE | 0.111 | 0.0267-0.196 | p＜0.001 |
| CNS-LAND vs THRIVE | 0.140 | 0.0443-0.236 | p＜0.001 |

CI indicates confidence interval
